# Tick populations and molecular detection of selected tick-borne pathogens in questing ticks from northern and central Tanzania

**DOI:** 10.1007/s10493-023-00816-0

**Published:** 2023-07-18

**Authors:** Isihaka Haji, Martin Simuunza, Ning Jiang, Qijun Chen

**Affiliations:** 1 Key Laboratory of Livestock Infectious Diseases in Northeast China, Key Laboratory of Zoonosis, Ministry of Education, Shenyang Agricultural University, Shenyang, 110866 China; 2 Department of Disease Control, School of Veterinary Medicine, University of Zambia, P. O. Box 32379, Lusaka, Zambia; 3 Africa Centre of Excellence for Infectious Diseases of Humans and Animals, University of Zambia, P. O. Box 32379, Lusaka, Zambia; 4 Research Unit for Pathogenic Mechanisms of Zoonotic Parasites, Chinese Academy of Medical Sciences, 120 Dongling Road, Shenyang, 110866 China; 5 Department of Veterinary Microbiology, Parasitology and Biotechnology, Sokoine University of Agriculture, P. O. Box 3019, Morogoro, Tanzania

**Keywords:** Ticks, Tick-borne pathogens, Epidemiology, Tanzania

## Abstract

Ticks are vectors and reservoirs of a variety of pathogens including protozoa, bacteria and viruses which cause tick-borne diseases (TBDs) in humans and livestock. TBDs pose serious constraints to the improvement of livestock production in tropical and subtropical regions of the world. Despite their wide distribution, information on the tick and pathogen relationship is scarce in Tanzania. We used nested PCR and sequencing to screen pathogens of public and veterinary health importance in ticks collected by flagging from four districts of Tanzania. In total, 2021 ticks comprising nine species were identified. DNA from ticks was pooled according to tick species, developmental stage, and location, then screened for *Babesia bigemina*, *Babesia bovis*, *Theileria parva* and *Coxiella burnetii*. Out of 377 pools, 34.7% were positive for at least one pathogen. *Theileria parva* was the most abundant with a minimum infection rate (MIR) of 2.8%, followed by *B. bigemina* (MIR = 1.8%) and *B. bovis* (MIR = 0.8%). Multiple pathogens detection was observed in 7.2% of the tested pools. However, PCR screening of individual tick DNA revealed that only 0.3% of the examined pools had co-infection. DNA of *C. burnetii* was never detected in any tick DNA pool. The MIR of tick-borne pathogens (TBPs) differed significantly among districts, seasons, tick species, and tick developmental stages. Sequence analysis showed that *B. bigemina* RAP-1a, *B. bovis* SBP-4, and *T. parva* p104 genes were conserved among pathogens in the four districts. Despite the absence of *C. burnetii* in ticks, considering its pathogenic potential, it is essential to continue monitoring for its possible recurrence in ticks. This information adds to the knowledge of TBPs epidemiology and will contribute to the scientific basis for planning future control strategies.

**Supplementary information:**

The online version contains supplementary material available at 10.1007/s10493-023-00816-0.

## Introduction

Ticks (Acari: Ixodoidea) represent a major threat to human and animal health worldwide due to their major role as vectors and reservoirs of a variety of zoonotic protozoan, bacterial and viral pathogens. Therefore, ticks play a critical role in maintaining tick-borne pathogens (TBPs) in nature (Bekloo et al. 2018). Tick-borne diseases (TBDs) impede the growth of the livestock sector and impose major constraints on the health and management of livestock in the tropic and subtropical regions globally (Jongejan and Uilenberg 2004). Severe effects of ticks and TBDs are observed mostly in rural populations where livestock is an essential source of income and food supply.

In East Africa, East Coast fever (ECF), babesiosis, and anaplasmosis are the major TBDs affecting livestock health and causing production losses (Ringo et al. 2020) whereas Q fever is among the most frequently reported tick-borne zoonoses (Crump et al. 2013; Njeru et al. 2016). ECF is a devastating disease associated with high mortality rates in cattle populations, caused by *Theileria parva* (Zobba et al. 2020) and transmitted by *Rhipicephalus appendiculatus* or *Rhipicephalus zambeziensis* ticks (Meneghi et al. 2016). The disease is prevalent in eastern, southern, and central Africa where its vectors are present (Adjou Moumouni et al. 2015).

In Tanzania and sub-Saharan Africa at large, bovine babesiosis is mainly caused by two distinct protozoa, *Babesia bigemin*a and *Babesia bovi*s (Lynen et al. 2008; Adjou Moumouni et al. 2015; Heylen et al. 2023). Bovine babesiosis is an acute disease that becomes persistent in animals that survive the infection and is characterized by fever, listlessness, anorexia, dehydration, and progressive hemolysis (Zintl et al. 2005). Whereas *B. bigemina* is transovarially transmitted by *Rhipicephalus* (*Boophilus*) *decoloratus* and *Rhipicephalus* (*Boophilus*) *microplus* ticks, *B. bovis* is efficiently transmitted only by *R.* (*B.*) *microplus*. Although *B. bigemina* is more widespread than *B. bovis*, the latter parasite is responsible for much heavier losses in susceptible livestock (Lynen et al. 2008).

*Coxiella burnetii* is an intracellular Gram-negative bacterium that occurs worldwide but not in New Zealand (Mediannikov et al. 2010). Its infection in humans and animals leads to Q fever and coxiellosis, respectively. Because of its potential for rapid spread and highly infectious nature, hence its effects on global public and veterinary health, *C. burnetii* has attracted significant attention for research purposes. Domestic animals such as sheep, goats, and cattle are considered the latent source of infection in humans. The infected animal sheds the bacterium through the placenta and amniotic fluids which may contaminate the environment (Maurin et al. 1999). The main transmission route of *C. burnetii* is via inhalation of contaminated particles. However, ticks are considered the natural primary vector of *C. burnetii* as they maintain the infection in domestic animals. Several tick species have been reported to carry natural infection and shed a significant number of viable *C. burnetii* in their feces (Maurin et al. 1999). This means, inhaling tick excreta can be a significant source of infection. Previous studies have confirmed the circulation of tick-borne zoonoses including *C. burnetii* in human and livestock samples in northern Tanzania (Crump et al. 2013)—despite this fact, no surveillance has been done to establish its prevalence in ticks.

Information on the infection rate of pathogens in ticks, and the genetic diversity of the circulating pathogens are important variables in understanding the epidemiology and control of TBDs. The risk of transmission of TBPs is determined by the prevalence of ticks in the environment and by the probability of an encounter between an infected tick and a susceptible host. Unfortunately, very few studies have identified TBPs of veterinary importance in ticks in Tanzania, and the majority of these few are using ticks collected while they are feeding on hosts. The procedure of measuring tick abundance and risk of pathogens using feeding ticks was challenged by Gray et al. (2021), because preferences for a given host and individual susceptibility of each vertebrate to carry ticks bias the data obtained. Estrada-Peña et al. (2021) emphasize the use of questing ticks to estimate the risk of pathogens as the reliable procedure that provides an unbiased view of the actual infection rates in the field.

Therefore, this study was carried out to determine and understand the epidemiology and genetic diversities of some TBPs circulating in ticks in Tanzania mainland using DNA-based PCR and sequencing.

## Materials and methods

### Description of the study area

Tick collection sites were located in Longido (2°42′S, 36°42′E), Gairo (6°14′S, 36°87′E), Mvomero (8°10′S, 28°37′E) and Monduli (3°20′S, 36°15′E) districts in Tanzania. Monduli and Longido districts are located in northern Tanzania. The area has a semi-arid ecosystem with average annual rainfall of 600–700 mm which falls mostly between March and May and in November and December. The climate is tropical sub-humid with average temperatures of 26 °C annually (Warwick et al. 2016).

Mvomero district has a bimodal rainfall distribution, with a long wet season from March to May and a short wet season from October to December. The climate is humid to sub-humid, annual rainfall ranges from 600 to 2000 mm. Average annual temperatures range from 20 to 30 °C. Gairo district is found in central Tanzania and experiences a total annual rainfall of 1200 mm, with a long rainfall season starting from December to February. The climate is tropical sub-humid with annual average temperature of 25–30 °C (Nonga et al. 2012).

### Collection of ticks from pastures

Questing ticks were collected from the pasture by dragging from February 2021 to October 2022. Dragging was performed along 90 line transects which were randomly selected (approximately 23 transects from each district). A white flag of 1 m^2^ was dragged along transect lines, averaging 90 m (Short and Norval 1981). After collection, the ticks were placed in 100 ml universal bottles with cotton wool dampened with sterile water. The bottles were placed in cool contained ice packs and transported to the laboratory for analysis. In each district, sampling was done twice (dry and rainy season).

### Tick identification and DNA extraction

The collected ticks were morphologically examined using a stereo microscope (80-fold magnification and identified to species level based on morphological features (Walker et al. 2014). DNA from individual ticks was extracted by the TIANamp Genomic DNA Kit (DP130227; Tiangen Biotech, Beijing, China) in accordance with manufacturer’s instructions. The DNA of each tick was divided into two equal volumes. One set of DNA was stored in Eppendorf tubes and frozen at − 20 °C until needed for further analysis. The second set of DNA was pooled into five according to tick species, developmental stage, and location of the collection.

### PCR amplification for detection of piroplasmids (*Babesia*/*Theileria*) and *Coxiella* like organisms

Each of the pooled tick DNA sample was screened with species-specific nested PCR (nPCR) primers (Table 1) for the presence of *B. bigemina* rhoptry-associated protein-1a (RAP-1a), *B. bovis* spherical body protein-4 (SBP-4), *T. parva* 104 kDa antigen (p104) and *C. burnetii* htpB genes (To et al. 1996; Skilton et al. 2002; Odongo et al. 2010; Terkawi et al. 2011). PCR amplification of both pathogens consisted of 16 µl of nuclease-free water which was added to a crystallized PCR premix (Accupower PCR Premix; Bioneer, South Korea), 2 µl of DNA template, and 1 µl (10 pmol) of each primer (total 20 µl reaction). Except for *C. burnetii* which used single PCR, 1 µl of DNA of each pathogen obtained from the first round of PCR was used as a template for the second round of nPCR, respectively.

**Table 1:** Sequences of primer sets used for detection of *Theileria parva*, *Babesia bovis*, *Babesia bigemina* and *Coxiella burnetii* DNA in tick pools

Pathogen	Assays	Oligonucleotide sequences (5′ > 3′)	Product size (bp)	References
*Theileria parva* p104	PCR	ATTTAAGGAACCTGACGTGACTGC	486	Skilton et al. (2002)
		TAAGATGCCGACTATTAATGACACC		
	nPCR	GGCCAAGGTCTCCTTCAGAATACG	277	Odongo et al. (2010)
		TGGGTGTGTTTCCTCGTCATCTGC		
*Babesia bovis* SBP-4	PCR	AGTTGTTGGAGGAGGCTAAT	907	Terkawi et al. (2011)
		TCCTTCTCGGCGTCCTTTTC		
	nPCR	GAAATCCCTGTTCCAGAG	503	
		TCGTTGATAACACTGCAA		
*Babesia bigemina* RAP-1a	PCR	GAGTCTGCCAAATCCTTAC	879	
		TCCTCTACAGCTGCTTCG		
	nPCR	AGCTTGCTTTCACAACTCGCC	412	
		TTGGTGCTTTGACCGACGACAT		
*Coxiella burnetii* htpB	PCR	GCGGGTGATGGTACCACAACA	501	To et al. (1996)
		GGCAATCACCAATAAGGGCCG		

Primary PCR amplifications of *T. parva*, *B. bigemina* and *B. bovis* were performed with an initial denaturation at 95 °C for 5 min, followed by 35 cycles of denaturation at 94 °C (30 s), annealing at 55 °C (1 min), and extension at 72 °C (1 min), followed by a final extension at 72 °C for 10 min. The cycling conditions for the second amplifications of *B. bigemina* and *B. bovis* were the same as that of primary amplification except for *B. bovis* whose annealing temperature was 50 °C. In the case of *T. parva*, the second amplification comprised of an initial denaturation at 95 °C for 5 min, 25 cycles of denaturation at 94 °C for 30 s, annealing at 58 °C for 1 min, extension at 72 °C for 1 min plus a final extension at 72 °C for 20 min. The PCR cycling condition of *C. burnetii* comprised 94 °C for 3 min, followed by 36 cycles of 94 °C for 1 min, 56 °C for 1 min, and 72 °C for 1 min, and then a final extension at 72 °C for 4 min. The reactions were performed using an automatic thermal cycler (Takara).

PCR products of each gene was visualized by UV light in a 1.5% agarose gel containing 3 µl GelRed (Biotium, Fremont, CA, USA). In the case of the pools which had multiple pathogens detection, nPCR of the respective individual ticks were performed using the stored set of individual tick DNA.

### Sequencing of the PCR-positive samples

In total, 13 samples (*B. bigemina*, n = 3; *B. bovis*, n = 5; and *T. parva*, n = 5) were randomly selected for DNA sequencing. Purification of the DNA of selected samples was done using Roche High PCR Purification Kit (Bioneer) as per the manufacturer’s protocol. The concentration of PCR products was checked by a Nanodrop system (Thermo-Scientific, UK). Each of the purified samples was sent to Macrogen (Europe) for automated nucleotide sequencing by Sanger dideoxy method with both the forward and reverse primers.

### Sequence and phylogenetic analyses

The returned sequences were edited in Geneious prime software v.2022.01 (created by Biomatters) (Kearse et al. 2012). Consensus sequences of each isolate were compared for identities and similarities to other published sequences available in GenBank using the basic local alignment search tool (BLAST) and comparison with sequences deposited in the non-redundant National Center for Biotechnology Information (NCBI) database (https://blast.ncbi.nlm.nih.gov/). Pairwise/multiple sequence alignments were done using CLUSTALX (Thompson et al. 1997). Aligned sequences were trimmed to the same length (with gaps) from which phylogenetic trees were constructed. Phylogenetic trees were constructed using MEGA v.11.0 (Tamura et al. 2021) with DNA sequences obtained from this study and those from the same pathogens already available in the GenBank. The evolutionary models for individual DNA sequence alignments were determined using the Akaike information criterion test in jModelTest v.3.7 (Darriba et al. 2012). Maximum likelihood method was used for phylogenetic tree analysis of *B. bovis* (SBP-4), *T. parva* (p104) and *B. bigemina* (RAP-1a) gene. The bootstrap consensus tree inferred from 1000 replicates (Tamura et al. 2004) was taken to represent the evolutionary history of the taxa analyzed.

### Statistical analysis

Pearson’s χ^2^ test was applied to analyze the MIR of each TBP according to independent variables such as tick developmental stages, tick species, season or study area, using SPSS v.22 (α = 0.05). The MIR of a pathogen was calculated by the following formula: MIR = X/(Y × Z) × 100%, where X is the number of positive pools, Y is the total number of pools tested and Z is the size of the pool. This formula assumes that only one tick is infected in a positive pool (Andreassen et al. 2012).

## Results

### Tick species identification

In total, 2021 hard ticks were collected within three tick genera namely *Rhipicephalus*, *Hyalomma*, and *Amblyomma*. The genus *Rhipicephalus*, with a prevalence of 82% (1658/2021), was the most prevalent, followed by the genus *Amblyomma* 14% (282/2021) and *Hyalomma* 4% (81/2021). In total, nine tick species were identified, and the most common was *R. appendiculatus* (n = 500, 24.7%), *R.* (*B.*) *decoloratus* (n = 428, 21.2%), *R.* (*B.*) *microplus* (n = 263, 13.0%), *A. variegatum* (n = 262, 13.0%), *R. evertsi evertsi* (n = 244, 12.1%), *R*. *pulchellus* (n = 172, 8.5%), *H. albiparmatum* (n = 81, 4.0%), *R. praetextatus* (n = 51, 2.5%) and *Amblyomma lepidum* (n = 20, 1.0%).

Tick species proportion varied among districts in the dry and wet seasons. Except for *R.* (*B.*) *decoloratus* (in Longido and Monduli) and *H. albiparmatum* (in Gairo and Monduli) which were more abundant in the dry season, and *R. praetextatus* which had relatively the same abundance (%) in the two seasons, all other tick species (˃ 70%) were more abundant in the wet season. However, the number of some species, such as *A. lepidum* collected in this study was too small to assess their seasonal dynamics. The details of the tick demographics are shown in Table 2.

**Table 2:** *Rhipicephalus* (*Boophilus*), *Amblyomma* and *Hyalomma* tick species collected from pasture during dry and wet seasons in Longido, Gairo, Mvomero and Monduli districts, Tanzania

Location (district)	Season	*R. appendiculatus*	*R. pulchellus*	*R.* (*B.*) *decoloratus*	*R.* (*B.*) *microplus*	*R. evertsi*	*R. praetextatus*	*A. variegatum*	*A. lepidum*	*H. albiparmatum*	Total
Longido	Dry	8	26	24	8	5	4	0	0	0	75
	Wet	134	27	0	0	6	7	8	0	0	152
Gairo	Dry	28	0	69	39	33	0	42	18	52	281
	Wet	64	0	97	51	95	0	38	0	25	370
Monduli	Dry	14	48	31	0	3	17	54	0	4	171
	Wet	116	71	11	0	15	23	69	2	0	307
Mvomero	Dry	24	0	74	55	33	0	33	0	0	219
	Wet	112	0	122	110	54	0	18	0	0	416
Total no. ticks (%)		500 (24.7)	172 (8.5)	428 (21.2)	263 (13)	244 (12.1)	51 (2.5)	262 (13)	20 (1)	81 (4)	2021 (100)

The proportion of tick developmental stages differed significantly among tick species and locations. *Rhipicephalus appendiculatus* had the highest (n = 195, 47%) percentage of nymphs collected, followed by *R. evertsi evertsi* (n = 80, 19%), *A. variegatum* (n = 65, 16%), *H. albiparmatum* (n = 30, 7%), *R*. *pulchellus* (n = 25, 6%), *A. lepidum* (n = 6, 1%) and *R. praetextatus* (n = 5, 1%). No nymphal stages of *R.* (*B.*) *decoloratus* or *R.* (*B.*) *microplus* were collected. The highest percentage of nymphs was found in Longido (54%) followed by Gairo (28%), Monduli (14%), and Mvomero (12%). The proportion of nymphal stages did not differ significantly among wet (21%) and dry (23%) seasons.

### Pathogens detected in the ticks and infection rates

*Theileria parva* was the most abundant (MIR = 2.8%), followed by *B. bigemina* (1.8%) and *B. bovis* (0.8%) (Table 3). DNA of *C. burnetii* was never detected in any tick pool.

**Table 3:** Minimum infection rates (MIR, %) of tick-borne pathogen DNA in pools of *Rhipicephalus* (*Boophilus*), *Amblyomma* and *Hyalomma* ticks (size n = 5 each) by PCR in Longido, Gairo, Mvomero and Monduli districts, Tanzania

Location (district)	Tick species	No. tick pools tested	No. positive pools (MIR %)					Total
			*Theileria parva*	*Coxiella burnetii*	*Babesia bigemina*	*Babesia bovis*	Co-detections	
Longido	*R.* (*B.*) *decoloratus*	3	0 (0)	0 (0)	2 (13.3)	0 (0)	0 (0)	2 (13.3)
	*R. appendiculatus*	28	5 (3.6)	0 (0)	0 (0)	0 (0)	6 (4.3) *T. parva* + *B. bigemina*	11 (7.9)
	*R.* (*B.*) *microplus*	2	0 (0)	0 (0)	0 (0)	0 (0)	0 (0)	0 (0)
	*R. evertsi evertsi*	1	0 (0)	0 (0)	0 (0)	0 (0)	0 (0)	0 (0)
	*R. praetextatus*	2	0 (0)	0 (0)	0 (0)	0 (0)	0 (0)	0 (0)
	*A. variegatum*	1	0 (0)	0 (0)	0 (0)	0 (0)	0 (0)	0 (0)
Total Longido		37	5 (2.7)	0 (0)	2 (1)	0 (0)	6 (3.2)	13 (7)
Gairo	*R.* (*B.*) *decoloratus*	33	0 (0)	0 (0)	16 (9.7)	0 (0)	7 (4.2) *T. parva + B. bigemina*	18 (13.9)
	*R.* (*B.*) *microplus*	18	0 (0)	0 (0)	0 (0)	11 (12.2)	1 (1.1) *B. bigemina + B. bovis*	12 (13.3)
	*R. appendiculatus*	18	3 (3.3)	0 (0)	0 (0)	0 (0)	0 (0)	3 (3.3)
	*R. evertsi evertsi*	25	1 (08)	0 (0)	0 (0)	0 (0)	0 (0)	1 (0.8)
	*A. variegatum*	16	0 (0)	0 (0)	0 (0)	0 (0)	4 (5) *T. parva + B. bigemina*	4 (5)
	*A. lepidum*	3	0 (0)	0 (0)	0 (0)	0 (0)	0 (0)	0 (0)
	*H. albiparmatum*	15	0 (0)	0 (0)	0 (0)	0 (0)	0 (0)	0 (0)
Total Gairo		128	4 (0.6)	0 (0)	16 (2.5)	11 (1.7)	12 (1.9)	43 (6.7)
Mvomero	*R.* (*B.*) *decoloratus*	39	0 (0)	0 (0)	10 (5.1)	0 (0)	0 (0)	10 (5.1)
	*R.* (*B.*) *microplus*	33	0 (0)	0 (0)	0 (0)	5 (3.3)	4 (2.4) *T. parva + B. bigemina + B. bovis*	9 (5.5)
	*R. appendiculatus*	26	17 (13.1)	0 (0)	0 (0)	0 (0)	0 (0)	17 (13.1)
	*R. evertsi evertsi*	17	4 (4.7)	0 (0)	0 (0)	0 (0)	0 (0)	1 (4.7)
	*A. variegatum*	10	0 (0)	0 (0)	0 (0)	0 (0)	0 (0)	0 (0)
Total Mvomero		125	21 (3.4)	0 (0)	10 (1.6)	5 (0.8)	4 (0.6)	40 (6.4)
Monduli	*R.* (*B.*) *decoloratus*	8	0 (0)	0 (0)	5 (12.5)	0 (0)	0 (0)	5 (12.5)
	*R. evertsi evertsi*	3	1 (6.7)	0 (0)	1 (6.7)	0 (0)	1 (6.7) *T. parva + B. bigemina*	3 (20)
	*R. appendiculatus*	24	18 (15)	0 (0)	0 (0)	0 (0)	2 (0) *T. parva + B. bigemina*	20 (16.7)
	*R. pulchellus*	23	4 (3.5)	0 (0)	0 (0)	0 (0)	0 (0)	4 (3.5)
	*R. praetextatus*	8	0 (0)	0 (0)	0 (0)	0 (0)	0 (0)	0 (0)
	*A. variegatum*	20	0 (0)	0 (0)	0 (0)	0 (0)	3 (3) *T. parva + B. bigemina*	3 (3)
	*H. albiparmatum*	1	0 (0)	0 (0)	0 (0)	0 (0)	0 (0)	0 (0)
Total Monduli		87	23 (5.3)	0 (0)	6 (1.4)	0 (0)	6 (1.4)	35 (8)
Overall total		377	85 (4.5)	0 (0)	34 (1.8)	16 (0.8)	28 (1.5)	131 (6.9)

The MIR of TBPs differed significantly among districts, seasons, tick species and tick developmental stages. Except for *B. bigemina* whose MIR did not differ among districts, the MIR of *T. parva* and *B. bovis* varied significantly. Higher MIR of *T. parva* was found in Mvomero (3.4%), Longido (2.7%), and Monduli (5.3%) than in Gairo (0.6%) district. *Babesia bovis* was detected only in Gairo (MIR = 1.7%) and Mvomero (0.8%).

*Babesia bigemina* was the only pathogen whose MIR varied significantly with the season and it was higher in the dry season (MIR = 3.4%) than in the wet (1%) season. The highest MIR of *T. parva*, *B. bigemina*, and *B. bovis* was found in *R. appendiculatus* (MIR = 6.5%), *R.* (*B.*) *decoloratus* (7.7%) and *R.* (*B.*) *microplus* (6.0%) ticks, respectively. Except for *T. parva* which was detected in both adult ticks and nymphs, the two species of *Babesia* were detected only in adult ticks of the subgenus *Boophilus*. A trend towards higher MIR was observed in adult ticks than in nymphs.

The details of how the MIR of each of the pathogens differed among various variables are shown in Table 4.. Representative images of PCR-positive gel electrophoresis for *B. bigemina*, *B. bovis*, *T. parva*, and *C. burnetii* are shown in Supplementary figures S1–S4.

**Table 4.** Minimum infection rates of *Theileria parva*, *Babesia bigemina*, and *Babesia bovis* for pools of *Rhipicephalus* (*Boophilus*), *Amblyomma* and *Hyalomma* ticks (size n = 5 each) by season, location, tick developmental stages and tick from Longido, Gairo, Mvomero and Monduli districts, Tanzania

Variable	No. pools for variable	No. positive pools (MIR %)			P
		*T. parva*	*B. bigemina*	*B. bovis*	
Season					
Wet	226	22 (2.9)	11 (1.)	14 (1.2)	*T. parva* = 0.45; *B. bigemina* = 0.001; *B. bovis* = 0.34
Dry	151	27 (2.7)	23 (3)	3(0.4)	
Location (district)					
Longido	37	5 (2.7)	2 (1.1)	0	*T. parva* = 0.003; *B. bigemina* = 0.41; *B. bovis* = 0.009
Gairo	128	4 (0.6)	16 (2.5)	11 (1.7)	
Mvomero	125	9 (1.4)	10 (1.6)	5 (0.8)	
Monduli	87	35 (20)	6 (1.4)	0	
Tick developmental stage					
Nymph	83	9 (2.2)	0	0	*T. parva* = 0.22; *B. bigemina* < 0.001; *B. bovis* = 0.017
Adult	293	44 (3)	34 (2.3)	16 (1.1)	
Tick species					
* R. appendiculatus*	96	31 (6.5)	0	0	*T. parva* < 0.001; *B. bigemina* < 0.001; *B. bovis* < 0.001
* R.* (*B.*) *decoloratus*	83	1 (0.2)	32 (7.7)	0	
* R.* (*B.*) *microplus*	53	0	1 (0.4)	16 (6)	
* A. variegatum*	47	7 (3)	0	0	
* R. evertsi evertsi*	46	4 (1.7)	1 (0.4)	0	
* R. pulchellus*	23	9 (7.8)	0	0	
* H. albiparmatum*	17	0	0	0	
* R. praetextatus*	8	1 (2.5)	0	0	
* A. lepidum*	4	0	0	0	

### Multiple pathogen detections

There were multiple pathogen detections, which involved double and triple infections, in 28 (7.4%) of the DNA pools (Table 3). However, PCR screening of the individual tick DNA revealed that only six (0.3%) of the examined ticks were co-infected. Four (66.7%) of the co-infections involved *T. parva* and *B. bigemina* in *R. appendiculatus* and *(A) variegatum* (nymph) from Gairo and Monduli districts. Two of the co-infections were of *(B) bigemina* and *B. bovis* in *R.* (*B.*) *microplus*, from Mvomero district.

### Comparative sequence analyses of piroplasmids (*Babesia*/*Theileria*)

All *B. bigemina*, *B. bovis* and *T. parva* sequences in this study were of the expected sizes. Nucleotide identity among the three *B. bigemina* (RAP-1a) gene sequences (ON221511–ON221513) was 99.7%. These sequences shared 99.5–100% nucleotide similarities with sequences from Tanzania (MG210824, MN 807,306), Uganda (MG426201, MG426202), and Turkey (KT220512). Furthermore, the percentage nucleotide similarities of all five *B. bovis* SBP-4 gene sequences (OM981234–OM981238) was 99.8% among themselves. These sequences showed 93.8–97.3% nucleotide similarities to sequences from Kenya (KP347555), Benin (KX685402), and Indonesia (KY484530).

The five *T. parva* (p104) gene sequences from this study (ON157060–ON157064) showed 99.6–100% nucleotide identity among themselves. These sequences had 97.8–99.6% nucleotide similarities with sequences from Uganda (MN810052), Tanzania (MN807321, MG700532), and Kenya (KP347566).

### Phylogenetic analyses

**Fig. 1:**
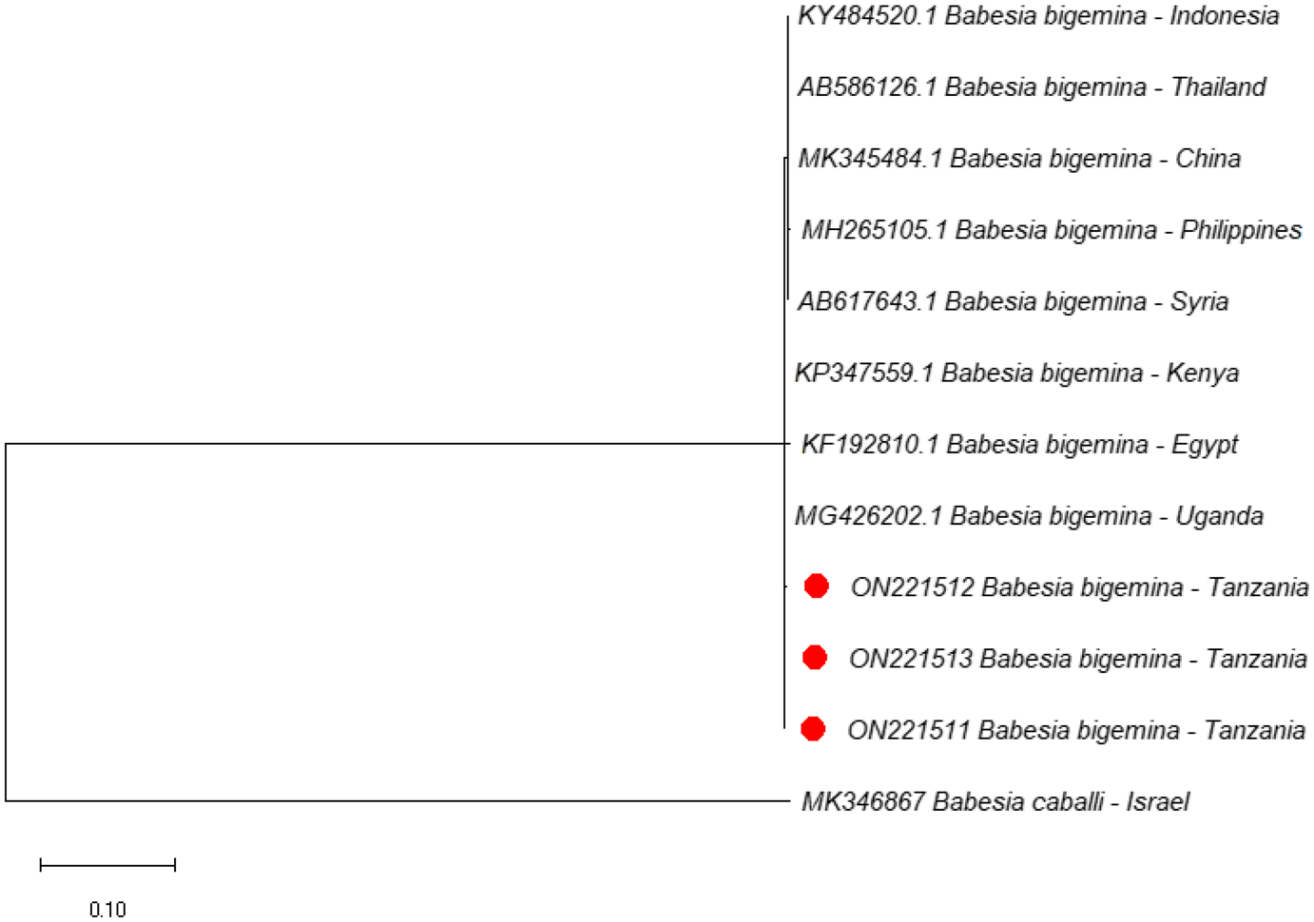
Phylogenetic analysis by maximum likelihood method based on *Babesia bigemina* RAP-1a gene sequences. Sequences from this study are marked with red dots. The RAP-1a gene of *Babesia bovis* (KT312810) was used as an outgroup

**Fig. 2:**
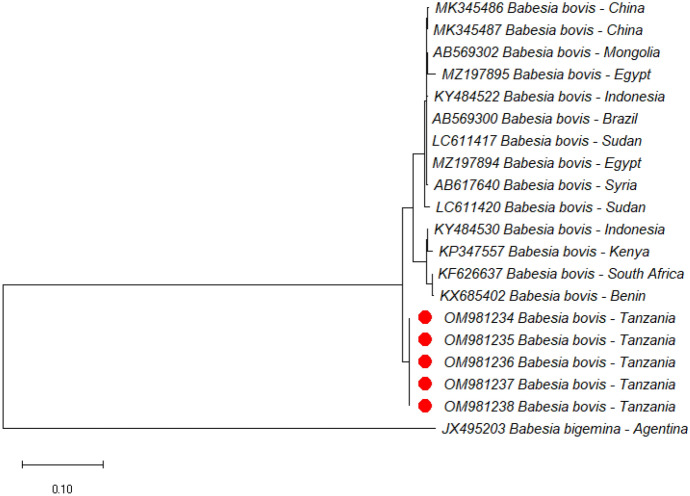
Phylogenetic analysis by maximum likelihood method based on *Babesia bovis* SBP-4 gene sequences. Sequences from this study are marked with red dots. The SBP-4 gene of *Babesia bigemina* (JX495203) was used as an outgroup

**Fig. 3:**
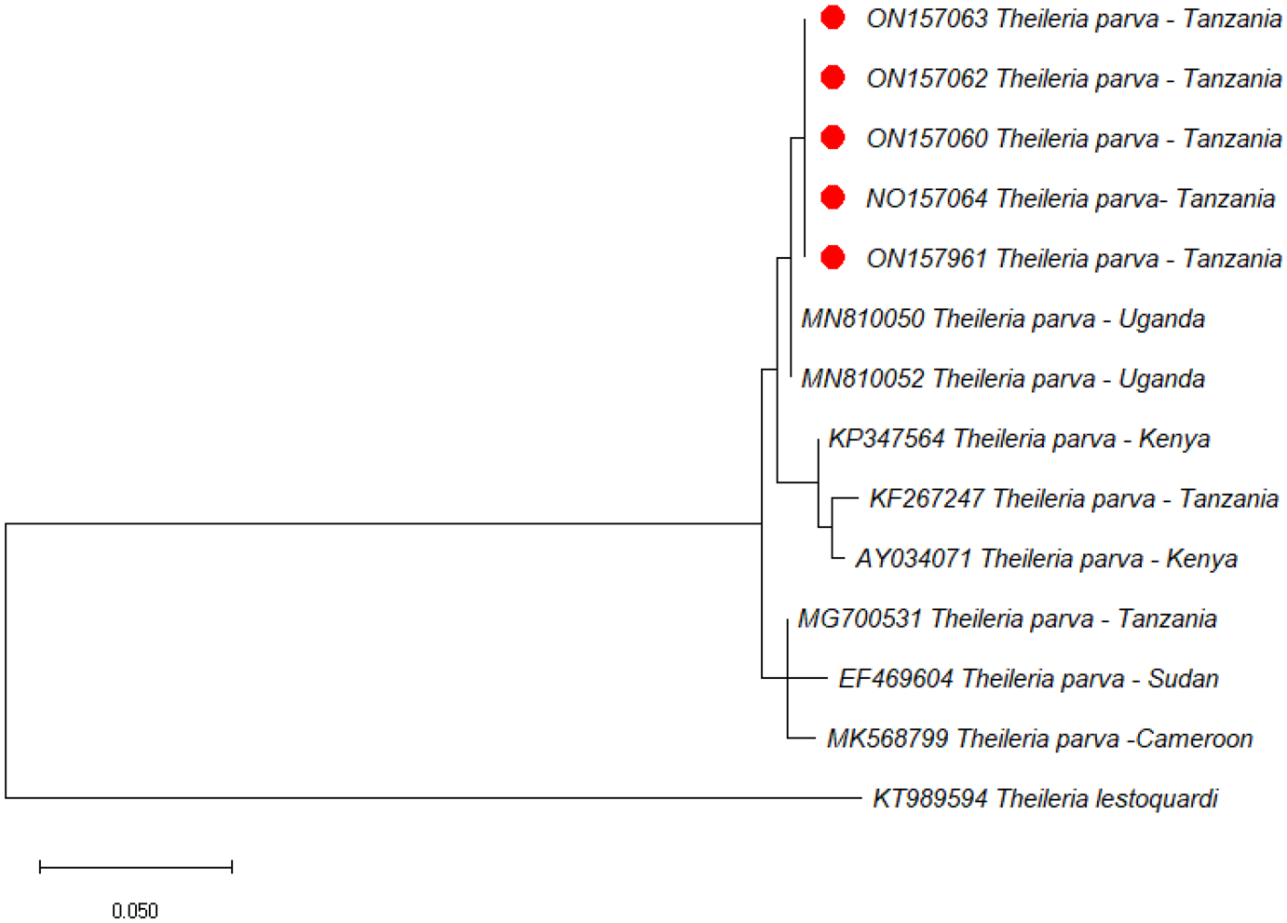
Phylogenetic analysis by Neighbor-joining method based on *Theileria parva* p104 gene sequences. Sequences from this study are marked with red dots. The p104 gene of *Theileria lestoquardi* (KT989594) was used as an outgroup

In the current study, phylogenetic trees of *B. bigemina*, *B. bovis* and *T. parva* were constructed based on RAP-1a, SBP-4, and p104 genes, respectively, using sequences from the NCBI GenBank. All three sequences of *B. bigemina* clustered in the same clade with sequences from Uganda (MG426202), Kenya (KP347559), and Egypt (KF192810) (Fig. 1). On the other hand, all the five SBP-4 sequences of *B. bovis* from this study clustered together forming a separate clade. Furthermore, five sequences of *T. parva* isolated in this study clustered together forming a separate clade but were close to other sequences from Uganda (MN810050 and MN810052). Other p104 sequences from Tanzania (KF267247 and MG700531) previously deposited in GenBank were placed in different clades. Representative images of the phylogenetic trees of *B. bigemina*, *B. bovis* and *T. parva* are shown in Figs. 1, 2 and 3.

The gene sequences produced from the current study were deposited in NCBI’s GenBank with the following GenBank accession numbers: ON221511–ON221513, ON157060–ON157064 and OM981234–OM981238.

## Discussion

This study reports tick species diversity and molecular detection of *B. bigemina*, *B. bovis* and *T. parva* in questing ticks as well as phylogenetic analysis of these pathogens but it could not detect *C. burnetii* from four districts of northern and central Tanzania. The presence of various tick species observed in the study sites may increase the risk of transmission of TBPs and the incidence of diseases in livestock and human populations. Some of the TBPs in the current study were detected in tick species that are not known to be their biological vectors. However, the detection of TBP DNAs in such tick species does not necessarily mean that the ticks can transmit the infection.

In line with previous reports (Nonga et al. 2012), this study indicates that *R. appendiculatus* is the dominant tick species in Tanzania. Furthermore, *R. appendiculatus* had the highest proportion of nymphs. The climate of these study areas is cool and moist, in such conditions *R. appendiculatus* reproduces most successfully as the climate is favorable to permit its cyclic activity and two consecutive generations can be completed in a year (Lynen et al. 2007).

*Rhipicephalus* (*B.*) *decoloratus* was the second most frequent species encountered in this study. A relatively high proportion of this tick species was in Gairo and Mvomero compared to Longido and Monduli districts. The high abundance of this tick species in these districts could be explained by Gairo and Mvomero having a conducive climate (> 800 mm rainfall annually) which favors the survival of this tick species relative to Longido and Monduli, which have a lower annual rainfall of 600–700 mm (Walker et al. 2014). Another species of the subgenus *Boophilus* observed in this study was *R.* (*B.*) *microplus*. However, this species was never collected in Monduli and only eight ticks were collected in Longido district. Furthermore, no nymphs of *R.* (*B.*) *decoloratus* / *R.* (*B.*) *microplus* were collected in this study. The absence of nymphal stages of these tick species can be attributed to these ticks being one-host ticks where once the larvae attach to the host they never drop to the vegetation until they become adults. This may have reduced the chances of collecting nymphs on the vegetation.

This study has recorded two *Amblyomma* species, *A. variegatum* and *A. lepidum*, the former being more prevalent than the later (14 vs. 1.0%). Unlike *A. lepidum*, which needs more specialized environmental conditions for survival, *A. variegatum* is the most widespread species among *Amblyomma* species covering the subhumid and low–high altitude areas of Tanzania (Lynen et al. 2007).

Tick population dynamics vary according to seasonal and location effects and have been linked to outbreaks of various TBDs (Lynen et al. 2008). Although many of the tick species in this study were found in both seasons, the total count of each species indicated a significant seasonal variation. As previously reported (Yawa et al. 2018), higher abundances were found in the wet than in the dry season. According to Walker et al. (2014), despite different tick species requiring different microclimates, most have higher reproduction activity during the wet season.

Although *T. parva* seems to have the highest MIR compared to other TBPs in this study, it is still low compared to that found in other studies (Bazarusanga et al. 2011) that used a similar pool size. In endemic areas like Tanzania where most of the infected ticks acquire infection from traditionally managed pastoral cattle, the lower rate of infection of *T. parva* in ticks is not uncommon (Swai et al. 2006), because host-to-tick transmission from traditionally managed animals is usually low (Medley et al. 1993). In addition, the grazing system in the study areas is free-range, where young cattle which usually have high parasitemia of *T. parva* are kept indoors in their first year of life to prevent contact with ticks and predators. As such, the ticks may feed only on low parasitemic adult-carrier cattle.

The MIR of pathogens detected in tick pools in the four study sites did not appear to differ in terms of numbers. However, the detection of *B. bovis* only in Gairo and Mvomero districts seems to correlate with the geographic abundance and distribution of its vector, *R.* (*B.*) *microplus* (a vector of both *B. bigemina* and *B. bovis*) which was mostly collected in the two districts. The only tick of the subgenus *Boophilu*s collected mostly in Longido and Monduli district was *R.* (*B.*) *decoloratus*, which can only efficiently transmit *B. bigemina* (Lynen et al. 2008).

Apart from the pathogens detected in this study, *A. variegatum* is the main vector of *Ehrlichia ruminantium* which causes heart water in cattle and *R. pulchellus* is a vector of *Theileria taurotragi*, the cause of benign bovine theileriosis. *Rhipicehalus evertsi evertsi* transmits *Anaplasma marginale*, the cause of bovine anaplasmosis, and can also release toxins that cause paralysis in cattle and sheep. Furthermore, *R. praetextatus* can transmit *Rickettsia conorii* and Nairobi sheep disease virus to humans and sheep, respectively (Walker et al. 2014).

The absence of *C. burnetii* DNA in all the ticks examined disagree with previous studies (Oswe et al. 2018) but corroborate Pilloux et al. (2018) who also reported a zero prevalence of the bacterium in tick pools. *Coxiella burnetii* is infrequently detected especially in flagging ticks (Knap et al. 2019). Although ticks play a minor role in Q fever transmission, it has also be noted that centers of attention in which ticks may act as the natural reservoir for *C. burnetii* seem to exist; only, these foci are hard to identify (Körner et al. 2021; Celina and Cerný 2022).

Co-infections of epidemiologically important pathogens in hard ticks have been previously reported and it varies primarily depending on geographic area and the number of pathogens screened (Rocha et al. 2022). This study has shown that 0.3% of the collected ticks were co-infected by various TBPs. In line with the previous reports (Moutailler et al. 2016; Klitgaard et al. 2019), which showed a higher percentage of co-infection in adult questing ticks than in questing nymphs, 83% of the co-infected ticks in this study were adult questing ticks. According to Rocha et al. (2022), adult ticks are more likely to be co-infected than nymphs because they may have had additional blood feeding. Ticks co-infected with multiple pathogens greatly increase the risk of co-infections in the vertebrate host, which would result in more complex clinical manifestations and could be misdiagnosed.

Results from the phylogenetic trees show that *B. bigemina* RAP-1a, *B. bovis* SBP-4 and *T*. *parva* p104 gene sequences isolated in the current study are conserved among *B. bigemina*, *B. bovis* and *T. parva*, respectively. These results disagree with Adjou Moumouni et al. (2015) from Kenya, where p104 genes of *T*. *parva* were clustered in different clades. The clustering of all the isolates of *B. bigemina*, *B. bovis* and *T. parva* from this study into their respective clades suggests their genetic relatedness. It is therefore possible that they may have a similar evolution despite coming from diverse geographical areas (Bekloo et al. 2018).

The major limitation with this study was the pooling of tick DNA samples instead of processing individual ticks. This was done mainly to avoid processing of large number of samples and also to reduce costs. As a result of this limitation, the multiple pathogens detected in a single tick pool did not necessarily mean that individual ticks were co-infected as it could also be caused by the simultaneous detection of several mono-infected ticks (AL-Hosary et al. 2021). To confirm this, all the tick pools with multiple infections were further screened individually using the second set of DNA to confirm the co-infection. However, despite this limitation, pooling of ticks for molecular detection of pathogens used in the current study is a useful common practice (Barghash et al. 2016), especially in resource-scarce settings (Speybroeck et al. 2012). Pooling of ticks for molecular detection of pathogens has also been previously used in different wild and domestic animals (Krishnamoorthy et al. 2021).

## Conclusion

This study revealed a diversity of tick species and TBPs affecting cattle in the study area. The infection rate of TBPs in ticks greatly differed among tick species, season, location, and tick developmental stages. *Theileria parva* was the most prevalent pathogens in questing ticks compared to the other detected pathogens (*B. bigemina* and *B. bovis*). These pathogens are phylogenetically similar among themselves, but differed from pathogens of other regions. The absence of *C. burnetii* in tick pools suggests an extremely low role of ticks as vector and reservoir of the bacterium in study areas. Nevertheless, considering its pathogenic potential, it is essential to continue monitoring for the possible recurrence of the bacterium in ticks in the future. This information adds to the knowledge of TBPs epidemiology in Tanzania and will be useful in the formulation of control strategies.

## Supplementary information

Below is the link to the electronic supplementary material.

Supplementary material 1 (DOCX 379.2 kb)

## Data Availability

Not applicable.
