# Supplementary material for: Tick populations and molecular detection of selected tick-borne pathogens in questing ticks from northern and central Tanzania
Source: Exp Appl Acarol. 2023 Jul 18;90(3-4):389–407. doi: 10.1007/s10493-023-00816-0 (PMC10406711; doi:10.1007/s10493-023-00816-0)
Supplement: Supplementary file 1 — Supplementary material 1 (DOCX 379.2 kb) [file 10493_2023_816_MOESM1_ESM.docx]

**Supplementary Materials**


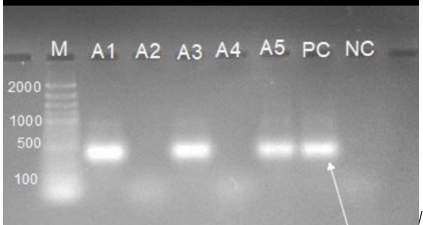


**Figure S1** Agarose-gel electrophoresis of nested polymerase chain reaction (nPCR) products amplified with genomic DNA from tick pools. M = 100-bp DNA marker. NC = negative control (nuclease free water), PC = Positive control (412 bp). Lanes A1, A3 and A5 are PCR products of samples positive for *Babesia bigemina*. Lanes A2 and A4 are PCR products of samples negative for *B. bigemina*.


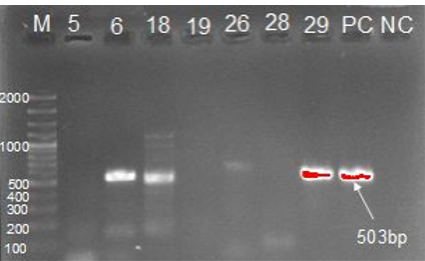


**Figure S2** Agarose-gel electrophoresis of nested polymerase chain reaction (nPCR) products amplified with genomic DNA from tick pools. M = 100-bp DNA marker. NC = negative control (nuclease free water), PC = Positive control (503 bp). Lanes 6, 18 and 29 are PCR products of samples positive for *Babesia bovis*. Lanes 5, 19, 26 and 28 are PCR products of samples negative for *B. bovis*.


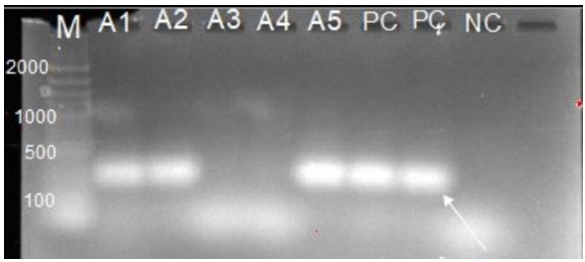


**Figure S3** Agarose-gel electrophoresis of nested polymerase chain reaction (nPCR) products amplified with genomic DNA from tick pools. M = 100-bp DNA marker. Lanes A1, A2 and A5 are nPCR products of samples positive for *Theileria parva*. Lanes A3 and A4 are PCR products of samples negative for *T. parva*. NC: negative PCR control (nuclease free water). PC: Positive control (277 bp).


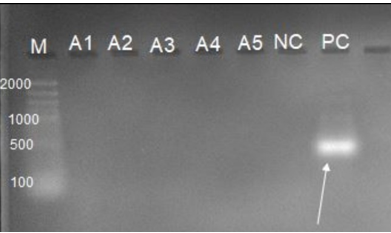


**Figure S4** Agarose-gel electrophoresis of polymerase chain reaction (PCR) products amplified with genomic DNA from tick pools. M = 100-bp DNA marker. NC = negative control (nuclease free water), PC = Positive control (501 bp). Lane A1–A5 are PCR products of samples negative for *Coxiella burnetii*.
